# Clinical significance of PET-CT coronary flow reserve in diagnosis of non-obstructive coronary artery disease

**DOI:** 10.1186/s13104-018-3667-0

**Published:** 2018-08-06

**Authors:** Henry Anselmo Mayala, Khamis Hassan Bakari, Fabian Pius Mghanga, Wang ZhaoHui

**Affiliations:** 1 Department of Cardiology 10th floor, Wuhan Union Hospital, Tongji Medical College of Huazhong University of Science and Technology, Internal medicine building number 1, Zhongshan avenue, Hankou, Wuhan, 43000 Hubei China; 2 Department of Radiology and Nuclear Medicine, Wuhan Union Hospital, Tongji Medical College of Huazhong University of Science and Technology, Wuhan, Hubei China; 3 Department of Internal Medicine, Archbishop James University College, Songea, Tanzania

**Keywords:** Coronary flow reserve, Coronary angiography, PET/CT, ECG, Coronary microvascular dysfunction

## Abstract

**Objective:**

To improve current knowledge of coronary flow reserve and non-obstructive coronary artery disease in terms of definition, features and clinical implications of measurement of coronary flow reserve (CFR), is an integrated measure of focal, diffuse, and small vessel coronary artery disease, can also be explained as a calculated ratio of hyperaemic to rest absolute myocardial blood flow. Non-obstructive coronary artery disease is defined as atherosclerotic plaque that does not obstruct blood flow or result in anginal symptoms. We also aimed at knowing the significance of PET in diagnosing coronary microvascular disease.

**Results:**

In our study 92% were between 41 and 60 years. 83% were males and 17% females, more patients had hypertension about 50%, few had diabetes mellitus about 16%, while those with both hypertension and diabetes mellitus were 17%. About 83% had ST segment and T wave changes on ECG. All patients and controls had normal coronaries on coronary angiography TIMI 3 flow. On further investigation by Positron emission tomography/CT we found out 58% had abnormal CFR and 42% had normal coronary flow reserve. Our findings indicate PET/CT coronary flow reserve concept provides a platform for the diagnosis of non-obstructive coronary artery disease in patients with signs and symptoms of ischemia without angiographic obstructive CAD.

## Introduction

Coronary flow reserve (CFR), is an integrated measure of focal, diffuse, and small vessel coronary artery disease, can also be explained as a calculated ratio of hyperaemic to rest absolute myocardial blood flow [1]. The coronary flow reserve (CFR) is a well validated index that allows the assessment of blood flow impairment originating from obstructive, diffuse, or microcirculatory involvement of the coronary circulation [2]. Diffuse coronary atherosclerosis is highly prevalent among patients with known or suspected coronary artery disease, increases the severity of inducible myocardial ischemia (beyond the effects of epicardial coronary obstruction), and identifies patients at high risk for serious adverse events, including cardiac death. Coronary flow reserve calculated as a ratio of hyperaemic to rest absolute myocardial blood flow (MBF) is a measure of coronary vasomotor dysfunction that integrates the hemodynamic effects of epicardial coronary stenosis, diffuse atherosclerosis, and microvascular dysfunction on myocardial tissue perfusion [3].

Most of the studies were done to study the epicardial coronary artery vessels and less focus on microvascular coronary vessels, that’s why we had to do this study.

## Main text

### Methodology

#### Study population

We assessed 12 patients admitted at department of cardiology, Wuhan union hospital, 10 who presented with typical angina symptoms and had a typical history of chest pain, ST-changes on EKG with normal coronary arteries on coronary angiography. 2 were control without any symptoms, no history of hypertension, diabetes mellitus or coronary artery disease, there ECG and CAG were normal.

#### Study type

Descriptive cross-sectional study.

#### Statistical analysis

Baseline patient characteristics were summarized. Statistical analyses were done using Graph pad prism version 7.04.

#### Ethics approval and consent to participate

This study was approved by the ethics committee of Tongji medical college of Huazhong university of science and technology. All the patients read and signed the informed consent form.

### Positron emission tomography

#### Image acquisition

All patients fasted for at least 6 h before PET/CT examination. The images were obtained using a dedicated PET/CT scanner (Discovery VCT^®^, GE medical systems, Milwaukee WI, USA) 40–60 min after intravenous injection of 3.75–5.55 MBq/kg of 18F-FDG. A low dose CT scan was obtained for attenuation correction using: tube voltage 120 kV, 80 mAs, and 3.75 mm slice collimation. PET data were constructed with the ordered subset expectation maximization algorithm. Both CT and PET data were sent to a work station (Xeleris^®^, GE medical systems) for evaluation.

PET-CT scan was used to measure coronary flow reserve and assess the microvascular coronary perfusion.

## Results

**Fig. 1:**
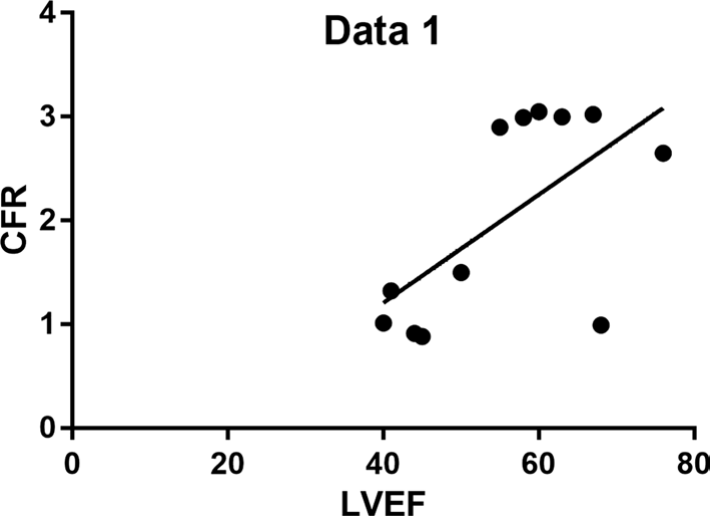
Fit plot showing a positive correlation between CFR and LVEF

**Fig. 2:**
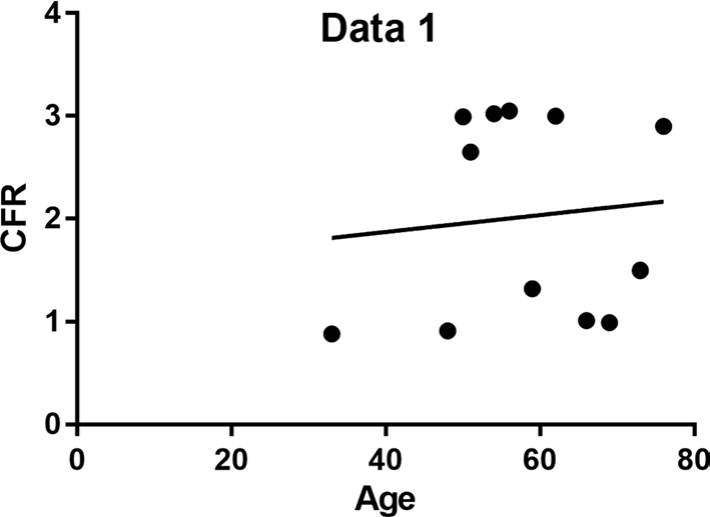
Fit plot showing no correlation between age and CFR

In our study we found that 8% were between the age of 21–40 years, and 92% were between 41 and 60 years. 83% were males and 17% females, more patients had hypertension about 50%, few had diabetes mellitus about 16%, while those with both hypertension and diabetes mellitus were 17% despite the above fact there those without hypertension and diabetes were also 17%. Furthermore, also few patients had Dyslipidemia about 17%, but most of them had ST and T changes on electrocardiogram about 83%, 67% had LVEF of more or equal to 50% while 33% had LVEF of less or equal to 50%, moreover 42% had regional wall abnormality picked on echocardiogram and 58% had normal regional wall motion on echocardiogram. All patients and controls had normal coronaries on coronary angiography TIMI 3 flow. On further investigation by Positron emission tomography/CT we found out 58% had abnormal coronary flow reserve and 42% had normal coronary flow reserve. We summarized some of the results on Figs. 1, 2 and Table 1.

**Table 1:** Patient characteristics

Parameter	Number of patients
Age	
21–40	1
41–60	11
Gender	
Male	10
Female	2
New York heart association (NYHA)	
I–II	9
III–IV	3
Comorbidities	
Diabetes mellitus	2
Hypertension	6
Diabetes mellitus + hypertension	2
Without any	
Dyslipidemia	
Elevated LDL-c	2
Electrocardiogram	
ST and T-changes	12
Echocardiogram	
LVEF ≥ 50%	8
LVEF ≤ 50%	4
Regional wall motion abnormality	5
Normal regional wall motion	7
Coronary angiography (CAG)	
TIMI III flow	12
PET	
Abnormal CFR (reduced CFR)	7
Normal CFR	5

## Discussion

Traditional approaches for risk assessment of ischemic heart disease are based on the physiological consequences of an epicardial coronary stenosis. Of note, normal coronary arteries or non-obstructive coronary artery disease is a common finding in women with signs and symptoms of ischemia [4], in our study we found out a fair number of males being affected by non-obstructive coronary artery disease about 86% of the affected, even though most of the study population were male, we wanted also to get a different perspective as more studies were done to female like a WISE study (Women Ischemia Syndrome Evaluation) [5–9], we also found out a fair number of patients had hypertension about 50%, few had diabetes mellitus 16 and 17% had both hypertension and diabetes mellitus, and those without hypertension and diabetes mellitus were 17%. What we have learned is that sometimes we need to think beyond our capacity in order to achieve the goal which is an accurate diagnosis, we have seen patients who presented with classical angina symptoms with ECG findings supporting ischemic changes but coronary angiography revealing TIMI III flow which means normal coronary flow, this means most of us clinicians when we get such results we believe the patient doesn’t have ischemic cardiac disease meaning no obstructive coronary artery disease but on further assessment and investigation with PET/CT scan we find out there is reduced coronary flow reserve reflecting the presence of non-obstructive coronary artery disease. There is one patient who had both risk factors hypertension and diabetes mellitus, but his CFR was normal but those who were smoking and had hypertension were mostly affected with an average CFR of 1.2, and there those who had no history of smoking, no hypertension and not diabetic but were symptomatic had a reduced CFR about 25%.

Coronary flow reserve is a non-invasive measure of coronary vasomotor function that integrates the hemodynamic effects of epicardial coronary stenosis, diffuse atherosclerosis and microvascular dysfunction on myocardial tissue perfusion [10] CFR can be measured non-invasively by PET, transthoracic Doppler echocardiography and cardiac MRI, in our study we chose PET because dynamic PET imaging affords robust and reproducible measurements of absolute myocardial blood flow (MBF) in ml/min/g at rest and during pharmacological stress which allows the calculation of CFR (defined as a ratio between MBF at stress and MBF at rest [10, 11]). For N-13 ammonia and Rb-82 tracers, CFR less than 2 is considered abnormal [12]. In our study we found out that 58% of our study population had reduced CFR of less than 2 of which they had normal CAG thus confirming the diagnosis of microvascular coronary artery disease and showing the high sensitivity of PET in diagnosing such condition supporting the literature.

Our study reveals the role of PET/CT in diagnosing microvascular coronary artery disease, irrespective of the natural history of the disease and other investigation findings, we should have high suspicion index on how to go further especially when we suspect the diagnosis of microvascular coronary artery disease, in so doing we will appropriately give the perfect management of such patients otherwise we might lose them, as they may have poor prognosis when they are not appropriately treated, as evidently in WISE study which showed that after 10 years follow up, cardiovascular death or myocardial infarction occurred in 6.7% of women with no evident coronary artery disease, and in 12.8% of those with non-obstructive coronary artery disease [13].

Hypertension and dyslipidemia are predictors of low coronary flow reserve irrespective of the concomitant effect of potential confounders. The CFR is often altered in arterial hypertension because of increased afterload or abnormalities of the LV structure (such as LVH) and function. The CFR is also reduced in patients with hyperlipidemia [14], this is in relation to our study were almost all patients with hypertension (n = 4 out of 5) 33.3% had low CFR and all patients who had dyslipidemia 16.6% had also low CFR thus concurring with the literature. We also found out all 41.6% of patients who had abnormal LV wall motion had low CFR, CFR is more sensitive than abnormal regional wall motion. However, the data for flow and function can be complimentary in terms of predicting underlying angiographic anatomy, because abnormal wall motion can include coronary artery disease and a normal CFR can exclude it [15].

## Conclusion

Our findings indicate PET/CT coronary flow reserve concept provides a platform for the diagnosis of non-obstructive coronary artery disease in patients with signs and symptoms of ischemia without angiographic obstructive CAD. This also provides the clinical significance of CFR as a diagnostic and prognostic indicator.

## Limitations

Our limitation mainly was small sample size, because of the cost of doing PET/CT which is still high.

## Abbreviations

CFR: coronary flow reserve
PET: positron emission tomography
CAD: coronary artery disease
